# Leveraging the Microbiome for Obesity: Moving From Form to Function

**DOI:** 10.3389/fendo.2022.918923

**Published:** 2022-07-07

**Authors:** Anna H. Lee, Amanda Manly, Tien S. Dong

**Affiliations:** ^1^ Department of Internal Medicine, University of California, Los Angeles, Los Angeles, CA, United States; ^2^ Department of Medicine, Garden City Hospital, Garden City, MI, United States; ^3^ Department of Gastroenterology, Greater Los Angeles Veterans Affairs, Los Angeles, CA, United States; ^4^ Vatche & Tamar Manoukian Division of Digestive Diseases, Los Angeles, CA, United States

**Keywords:** gut, microbiome, microbiota, obesity, weight

## Abstract

Treatment of obesity, an ongoing global epidemic, is challenging, as weight-loss efforts require a multidisciplinary approach addressing both behavioral and biologic needs that are not completely understood. Recent studies of the gut microbiome may provide better insight into the condition, and ultimately serve to advance more effective therapies. Research in this field has shifted from analyzing microbiome compositional differences to investigating functional changes that affect disease pathophysiology and outcome. Bacteria-derived metabolites are a way to bridge compositional changes to functional consequences. Through the production of metabolites, such as short chain fatty acids, tryptophan derivatives and bile acids, and interactions with peripheral and central signaling pathways, the gut microbiome may alter the body’s metabolic and behavioral responses to food. Here, we summarize these mechanisms driven by gut-derived metabolites, through which the microbiome is thought to contribute to obesity, as well as review recent investigations of interventions related to these metabolites. Limitations of existing research, primarily due to paucity of causal studies in humans, are also discussed in this review.

## Introduction

**Figure 1:**
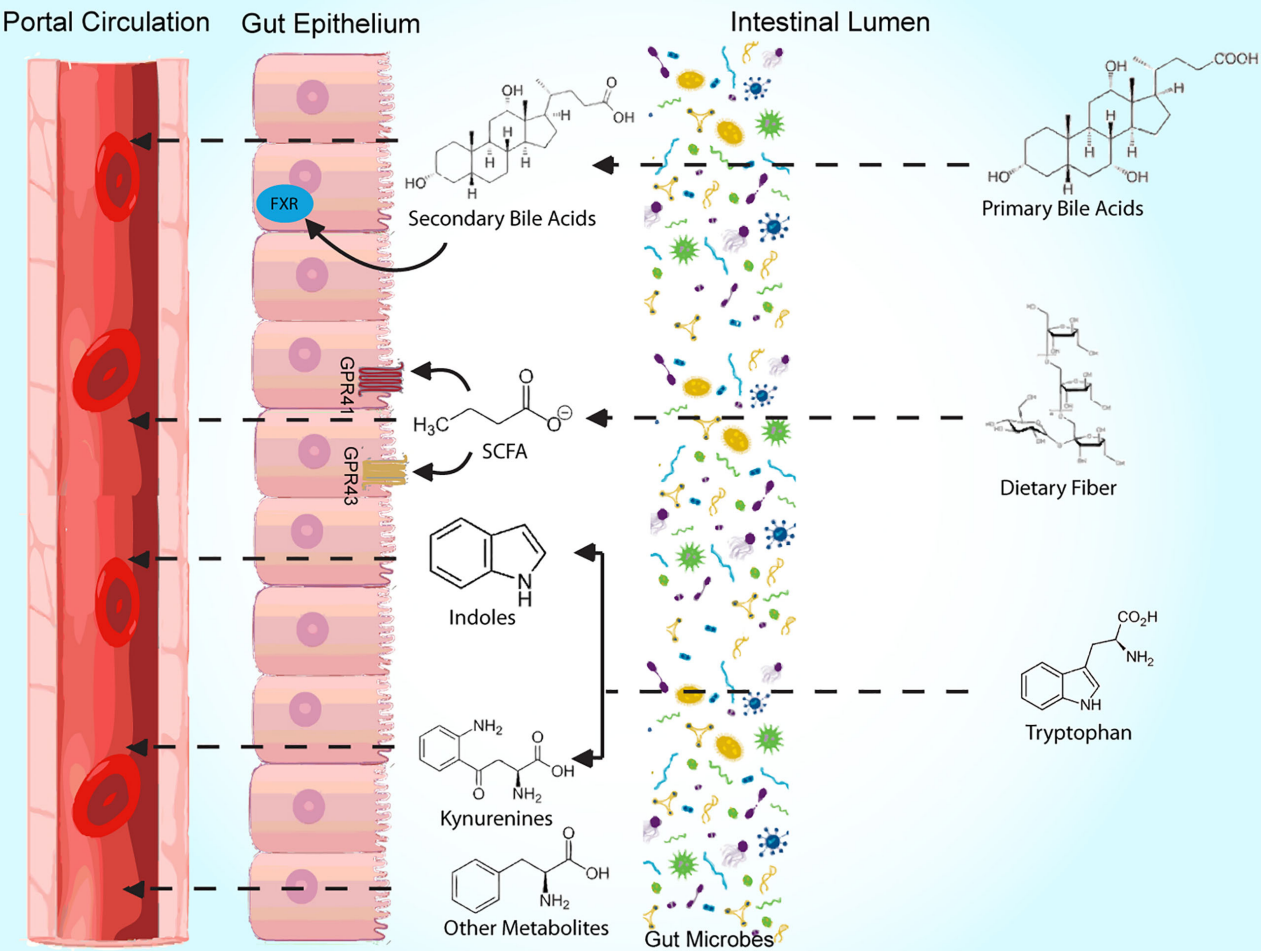
Summary diagram of bacterial metabolites and some of their mechanisms of action.

The obesity epidemic, which affects greater than a third of Americans (1), is a global health issue involving 650 million adults worldwide (2). Successful treatment of obesity has historically been difficult (3), likely from insufficient knowledge of its pathophysiology, and microbial colonizers of the gut, which have gained recognition for their role in metabolic disease, may serve as the missing link. Early research of the gut microbiome’s role in weight regulation has largely involved correlational studies of microbial composition, and differences in microbiome content have been reported among groups with varying genetic and environmental backgrounds (4, 5). Further, studies have associated obesity with taxonomic changes of the microbiome in response to antibiotic use (6, 7); similar investigations of the microbiome have been performed to evaluate various dietary interventions (8–11). However, in recent studies, there has been a shift prioritizing assessment of microbial function over composition in an effort to better guide its use in treatment of obesity. In this review, we summarize key and recent literature related to mechanisms of several commonly studied metabolites involved in microbiome-mediated pathways resulting in obesity, including short chain fatty acids, bile acids, and tryptophan derivatives, in obesity development (**Figure 1**, **Table 1**). Clinical applications, as they relate to these metabolites, are also discussed. We searched for original research articles in PubMed and Google Scholar using combinations of the following key words: gut microbiome, obesity, short chain fatty acid, bile acids, tryptophan, LPS.

**Table 1:** Summary table of mechanisms and downstream effects of each metabolite.

Metabolite	Target*	Proposed Downstream Effects	Effect on Weight
Short Chain Fatty Acids	(+)GPR43 (FFAR1) (+)GPR41 (FFAR3)	• GLP-1 and PYY release, leptin mRNA expression • Increase adipocyte oxidation (e.g., lpl, fiaf) and adipose tissue beiging	↓
	(–)HDAC	• Antioxidation (e.g., sod2, catalase) • Decrease inflammation (e.g., ccl2) • Increase mitochondrial synthesis (e.g., pgc1a)	↓
Bile Acids	(–)Intestinal FXR	• Regulate bile acid synthesis *via* FGF15 • Increase adipose tissue thermogenesis (e.g., ucp1, pgc1a, cox7a)	↓
	(+)Intestinal FXR	• Regulate bile acid synthesis *via* FGF15 • GLP-1 release • Activate TGR-5 • Increase adipose tissue browning, insulin sensitivity, glycemic control	↓
	(+)TGR5	• GLP-1 release	↓
Tryptophan Derivatives (Indoles)	(+)AhR	• Decrease inflammation (e.g., TNFα, IFγ), LPS translocation and gut permeability • Increase GLP-1 gene expression in intestines	↓
Lipopolysaccharides (LPS)	(+)TLR2 (+)TLR4	• Increase inflammation in white adipose tissue (e.g., ccl2, TNFα)	↑
Polyunsaturated Fatty Acids	(+)GPR40 (+)GPR120	• Decrease LPS-induced inflammation systemically and in hypothalamus (e.g., TNFα-induced inflammation, TLR-2 and TLR-3 inhibition) • Regulate adipogenesis (e.g., me1) • Improve insulin sensitivity (e.g., GLUT4 translocation in adipocytes and glucose transport)	↓

*(+) activation of (–); inhibition of

AhR, aryl hydrocarbon receptor; ccl2, C-C motif chemokine ligand 2; cox7a, cytochrome c oxidase subumit 7a; FFAR, free fatty acid receptor; FGF15, fibroblast growth factor 15; fiaf, fasting-induced adipocyte factor; FXR, farnesoid X receptor; GLP-1, glucagon-like peptide 1; GLUT4, glucose transporter type 4; GPR, G-protein coupled receptor; IFγ, interferon gamma; HDAC, histone deacetylase; lpl, lipoprotein lipase; me1, cytosolic malic enzyme 1; pgc1a, peroxisome proliferator-activated receptor gamma coativator-1 alpha; PYY, peptide YY; sod2, superoxide dismutase 2; TGR, Takeda G-protein coupled receptor; TLR, toll-like receptor; TNFα, tumor necrosis factor alpha; ucp1, uncoupling protein 1.

## Mechanism of Action

### Short Chain Fatty Acids

The most abundant microbial metabolites are short-chain fatty acids (SCFAs), which are breakdown products of carbohydrates that occur from bacterial fermentation in the gut. Numerous studies have investigated the role of various SCFAs in mediating the gut microbiota’s effects on metabolic syndrome. In general, SCFAs have been associated with beneficial effects on metabolic health. For instance, higher levels of fecal SCFAs, including butyrate, acetate, and propionate, in humans have been correlated with markers indicative of improved insulin resistance, obesity and food intake (12–14).

To better understand these gut-derived metabolites, studies have investigated the role of G protein-coupled receptors, namely GPR43 and GPR41 (alternatively known as free fatty acid receptors 1 and 3, respectively), which are both activated by SCFAs (15). Kimura *et al.* found that mice with knockout genes (KO) for GPR43 gained weight without a high fat diet (HFD), while GPR43 overexpression prevented obesity despite a HFD (16). Results from such studies serve as evidence of the receptor’s involvement in obesity development, which may in part be mediated by the release of satiety hormones glucagon-like peptide 1 (GLP-1) and peptide YY (PYY) from enteroendocrine cells (17), free fatty acid oxidation of adipose tissue (18), and control of energy expenditure (16). Additionally, GPR41 expression on vagal sensory neurons suggests that the receptor may influence centrally mediated effects on normal eating behavior, which was altered in GPR41 KO mice (19). SCFAs may also directly communicate with the central nervous system, as evidenced by carbon-labeled uptake by the brain of intraperitoneally administered acetate in a PET-CT imaging study (14).

Alternatively, SCFAs may act by inhibiting histone deacetylase (HDAC), a crucial enzyme in DNA transcription, given reduced HDAC activity measured in butyrate-treated enterocytes (20). Further, reduction of weight gain with butyrate treatment in HFD-fed mice was lost in HDAC KO mice, highlighting the significance of this enzyme in mediating SCFA’s effects (20). Butyrate-mediated HDAC inhibition has been associated with increased gene expression for PYY (21), as well as for various antioxidants and mitochondrial synthesis (22), which reduce metabolic dysfunction. In summary, SCFA’s favorable effects on obesity are likely channeled through several pathways.

Of note, data supporting beneficial effects of SCFAs have been met with some skepticism, as results from an early study of obese mice were suggestive of greater fecal energy extraction in association with higher levels of SCFAs (23). In subsequent studies, higher fecal concentrations of both various and total SCFAs have been linked to obesity (24–26), and further, circulating levels of SCFAs have been positively associated with weight (27, 28). More studies are needed to elucidate the mechanisms and clinical effects of SCFAs.

### Bile Acids

The function of bile acids (BAs) within the gut not only lies in intestinal lipid absorption, but also in mediating the metabolic effects of the microbiome. From cholesterol, primary BAs are formed and subsequently conjugated in the liver; when released into the gut, microbiota mediate deconjugation and metabolism to secondary BAs (29). In a correlational study of microbiome from both mice and human subjects, levels of the BAs ursodeoxycholate (UDCA), chenodeoxycholate, and lithocholate (LCA) were reduced in obesity (30), suggesting beneficial effects of non-12-hydroxylated BAs. Similarly, mice that had improvements in metabolic markers, such as weight, after *Parabacateroides distasonis* administration that elevated UDCA and LCA (31). Further, antibiotic treatment leading to altered microbiome in mice produced both an improved metabolic phenotypes and increased tauro-β-muricholic acid (TBMCA) levels (32).

BA mediated outcomes on metabolic syndrome involve the farnesoid X receptor (FXR), whose activation modulates expression of genes regulating metabolism and BA synthesis (33, 34). FXR antagonism by BAs such as glycine-β-muricholic acid (Gly-MCA) and TBMCA in intestinal cells has been associated with prevention of obesity (32, 35), with similar effects seen from mice with intestinal FXR KO genes (36). The microbiome’s link to this pathway was strengthened in a study demonstrating more obesity in conventionally raised, compared to GF mice but no difference in weight when mice were FXR KO (36). However, intestine-specific FXR agonists such as fexaramine also generated favorable metabolic profiles that were associated with BA compositional changes (37–39). These mixed results of FXR-mediated activity are not completely understood and require further investigation.

In addition to FXR, Takeda G-protein coupled receptor 5 (TGR5) is also essential in the BA pathway, where its agonism and overexpression in HFD-fed mice were both linked to metabolic improvement (40). These outcomes may result from TGR5-stimulated release of GLP-1, and receptor activation likely plays a role in both peripherally-mediated metabolic and centrally-mediated eating/behavioral mechanisms influencing obesity (40–42). Effective use of BAs in combating obesity may need to involve both receptors, which have been shown to produce distinct downstream effects from one another (43).

### Tryptophan Derivatives

In addition to its many roles as an essential amino acid, tryptophan also modulates the microbiome’s effects on weight and metabolism (44). After dietary ingestion, processing by the gut can follow one of three pathways forming either serotonin, kynurenine (Kyn) or indole metabolites (44). The link between tryptophan derivatives and the gut microbiome was strengthened in several correlation studies (45), including one that associated changes in microbial composition with levels of tryptophan-derived neurotransmitters and neurotransmitter transporters in piglets administered antibiotics (46). Further, the presence of spore forming bacteria were linked to higher levels of serotonergic metabolites in mice (47). Obesity development has also been attributed to activity of indoleamine 2,3 dioxygenase (IDO), yielding higher levels of kynurenine, as well as with lower circulating levels of indole products (48–50). When mice on a HFD were either administered indole or genetically lacked IDO, weight gain was mitigated (48, 50).

Trypophan-derived metabolites likely exert their effects in part through aryl hydrocarbon receptors (AhR) (33, 51), given positive associations seen among indole metabolites, AhR activation and improved metabolic markers. For instance, diet induced obese (DIO) mice that had lower fecal indole derivative concentrations also had decreased AhR activity, and several metabolic effects (such as insulin sensitivity) were reversed with use of an AhR agonist (52). Effects are thought to be mediated by the microbiome, as indole metabolite levels were altered in conventional, but not in GF, mice on HFD (53). However, AhR antagonism was also associated with beneficial metabolic effects in a study using both ligands antagonizing AhR and AhR genetic deletions in mice (54); given that this inconsistent response was thought to be mediated by Kyn rather than indole, future studies may be needed to evaluate differences in response from various AhR ligands.

Downstream effects of microbial tryptophan metabolism have been correlated to the central nervous system as part of the brain-gut axis. In trials of obese humans, indolepropionic acid (IPA) levels inversely correlated with food addiction and magnetic resonance imaging (MRI) derived activity in reward centers of the brain (55), with the latter finding seen again in relation to fecal tryptophan levels (56). In addition, central inhibitory control positively correlated with IPA levels in obese humans; mice with microbial transplants from obese humans with impaired inhibitory control not only exhibited similar behavior but also altered prefrontal cortex activity as measured by metabolic gene expression (57). Tryptophan and its metabolites, as part of the brain-gut axis, have gained recognition for their integral role in obesity development.

### Miscellaneous Metabolites

Metabolic influence of the gut microbiome may also involve the immune system. In a study of rats continuously infused lipopolysaccharide (LPS), often a virulent component of gram-negative bacterial cell walls (58), chronic LPS exposure led to hyperphagia and leptin resistance (59). However, virulence and endotoxic response from LPS may vary, contingent on the bacterium from which it is derived (60). Toll-like receptors (TLR) may mediate these effects. Caesar *et al.* reported greater weight gain and activation of LPS receptor, TLR4, from serum of lard-fed mice, with weight preservation in mice with KO genes for TLR adaptor molecules (61). Further, serum bacterial DNA levels were similar between obese and nonobese mice, suggesting a more direct role for molecular signaling than systemic bacterial infiltration in inducing these effects (61). TLR4 activation is linked to increases in inflammatory markers within adipose tissue, which in turn produces phenotypes associated with metabolic syndrome (61).

Polyunsaturated fatty acid (PUFA)-derived metabolites have also been linked to metabolic health. The microbiome was implicated in transforming PUFAs to their metabolites in a study that reported lower fecal levels of PUFA metabolites in GF, compared to conventional, mice (62). Further, FMTs from HFD-fed mice supplemented with a PUFA metabolite enhanced glycemic control compared to those from mice without supplementation (63). These gut-derived substances may act through their activation of GPR40 and GPR120, producing favorable outcomes such as reduction of inflammation, lipogenesis, and glucose intolerance (64–66). Overall, results from studies involving these metabolites have been encouraging.

## Clinical Applications

In this section, we discuss potential novel options for oral treatment of obesity involving the gut microbiome, in the context of alterations to metabolites and mechanisms discussed in our review.

### Direct Microbiome Involvement

Two of the most common classes of therapies targeted directly at changing composition of the gut include prebiotics and probiotics. Though outcomes from studies measuring probiotic efficacy in treating obesity have been mixed, recent clinical trials of *Akkermansia muciniphila* have shown promising results. Obese patients who received *A. muciniphila* supplementation achieved more weight loss compared to those who received placebo, and metabolic changes in the treatment group were associated with decrease in plasma LPS levels; however, a difference was not seen with levels of GLP-1 (67). Mouse organoids exposed to *A. muciniphila* experienced greater modulation of genes, such as HDAC and Gpr43, than those for *Faecalibacterium prausnitzii* (68), which may suggest more efficacy of *A. muiniphila* use. In addition, various prebiotics, which are fibers digested by the gut microbiome, have been associated with changes in microbial composition (69–71), as well as with weight and metabolic activity (70, 71). Despite some encouraging data, no formal recommendations on use of *A. muciniphila* or prebiotic formulations exist.

Polyphenols are plant metabolites often poorly absorbed in the gut, resulting in delivery to gut microbiome for processing and essentially functioning as prebiotics (72). Several studies of polyphenols, such as resveratrol and various fruit extracts have correlated levels with improved metabolic markers (73–75) and altered microbial bile acid composition, TGR5 expression and TLR4 activation (76, 77), indicative of microbiome-induced metabolic changes. However, poor bioavailability has limited its clinical applicability, and additional studies of human use are warranted to develop effective formulations of polyphenol ingredients (78, 79).

### Indirect Microbiome Involvement

Other potential therapies are aimed at pathways involving the gut microbiome. For example, synthetic forms of metabolites from microbial processing have been studied in instances when poor drug delivery restricts effective use. Clinical use of butyrate is limited by its short half-life; instead, its prodrug, tributyrin may provide metabolic benefit with an improved pharmacokinetic profile (80). Additionally, Gly-MCA, bile acid derivative, effectively inhibited intestinal FXR to reduce obesity in mice, while resisting hydrolytic activity by bile salt hydrolase, whose activity could hinder its use *in vivo* (35). Alternatively, treatment with obeticholic acid, an FXR agonist with better delivery compared to its less lipophilic bile acid derivative, has been associated with improved hepatic steatosis in humans (81, 82).

Therapeutic agents for obesity may also target associated pathways of gut-microbial action. Use of BA sequestrants reducing intracolonic BA levels may have downstream, suppressive activity on FXR and has been linked to GLP-1 secretion and improved glycemic control (83). Perhaps the most well-recognized instance of microbiome-induced pathway modulation involves the use of GLP-1 receptor agonists, such as semaglutide (84) whose success and widely accepted use in both diabetes and obesity treatment is unsurprising, given the extent of data associating GLP-1 release with healthier phenotypes. In summary, modulations to microbial metabolites and their complex pathways present considerable opportunities for obesity treatment, which require further investigation.

## Discussion and Limitations

As we deepen our understanding of the gut’s role in metabolism and advance our knowledge of precision medicine, it becomes more evident that incorporating the microbiome will be critical to both prognostication and treatment of obesity. While recent research has focused on characterizing function over composition of the microbiome, an interplay between the two inherently exists, and treatment should integrate knowledge of both entities. For instance, we believe that microbiome-based therapies will be an adjunct to established treatments for obesity, as well as a tool for personalized medicine in the future. Oral probiotic or prebiotic supplements aimed at altering the composition and function of the microbiome, in conjunction with dietary and lifestyle modifications, can reach separate targets and increase the likelihood of success for weight loss. Furthermore, systemic and fecal concentrations of molecular signals from the microbiome’s functional domain may serve as biomarkers to predict outcomes, including risk of obesity, development of metabolic syndrome, or likelihood to respond to therapies for obesity, such as specialized diets (e.g., keto, Mediterranean, high protein) or bariatric surgery.

While the gut microbiome holds promise in improving treatment options for obesity, several challenges remain. A significant proportion of data stems from studies involving mice, which may be less accurate when applied to humans (85). Further, trials involving human subjects may also present difficulties. First, variations in human behavior may restrict the ability to conduct controlled studies. For example, compliance with an intervention such as diet or exercise may vary between subjects and often relies on the participant’s disclosure, which may be inaccurate. Given the impact of patient-dependent factors, such as diet, on the gut microbiome, a wide spectrum of baseline microbial composition and diversity likely exists, which may affect outcomes. Additionally, unlike studies of GF and genetically altered mice, trials involving humans have mostly produced data suggestive of correlational, rather than causative, relationships.

There are also limitations to the existing knowledge in the field. Well-defined roles for specific microbes of the gut have yet to be defined, and there are varied results in the literature. This is also true of several molecules, such as FXR and AhR, whose activation has been associated with both beneficial and harmful effects on metabolic disease. We may still have limited understanding of complex signaling pathways, which should continue to be investigated. Further, there should be increased efforts to evaluate and discover clinical applications of the gut microbiome in obesity treatment.

In summary, a rapidly expanding body of research suggests that the gut microbiome is essential in the development of metabolic diseases, including obesity. This is likely mediated through several gut-derived metabolites and their downstream effects on both central and peripheral pathways, which require further research and understanding.

## Author Contributions

AHL: literature review, manuscript preparation. AM: literature review, manuscript preparation. TD: manuscript revisions and revision, tables. All authors contributed to the article and approved the submitted version.

## Conflict of Interest

The authors declare that the research was conducted in the absence of any commercial or financial relationships that could be construed as a potential conflict of interest.

## Publisher’s Note

All claims expressed in this article are solely those of the authors and do not necessarily represent those of their affiliated organizations, or those of the publisher, the editors and the reviewers. Any product that may be evaluated in this article, or claim that may be made by its manufacturer, is not guaranteed or endorsed by the publisher.
